# Supplementary material for: Cationic amino acid transporters play key roles in the survival and transmission of apicomplexan parasites
Source: Nat Commun. 2017 Feb 16;8:14455. doi: 10.1038/ncomms14455 (PMC5316894; doi:10.1038/ncomms14455)
Supplement: Supplementary Information — Supplementary Figures, Supplementary Table, Supplementary References. [file ncomms14455-s1.pdf]

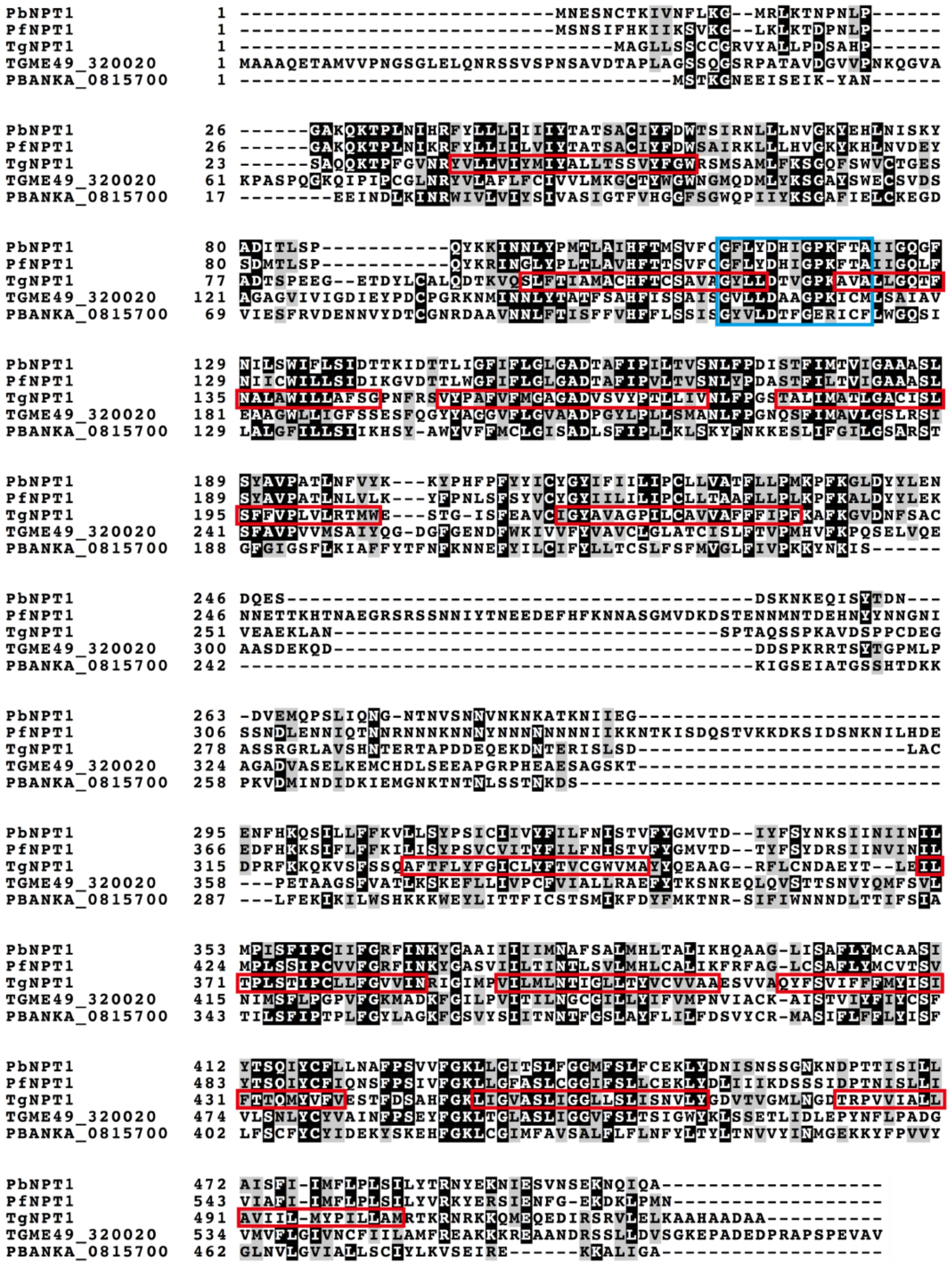

Supplementary Figure 1. Sequence alignment of representative members of *T. gondii, P. berghei* and *P. falciparum* NPTs. The twelve predicted transmembrane domains of *Tg*NPT1 are marked in red. The black shading indicates amino acid positions in which the residues in ≥3 of the 5 sequences are identical; the grey shading indicates those amino acid positions in which the residues in ≥3 of the 5 sequences are similar. The partially conserved Major Facilitator Superfamily (MFS) signature motif (G-[RKPATY]-L-[GAS]-[DN]-[RK]-[FY]-G-R-[RK]-[RKP]-[LIVGST]-[LIM]; ref 1), found between transmembrane domains 2 and 3 of this family of proteins, is marked in blue, and is typically G-X-L-L-D-X-X-G-P-K-X-X-X in NPT family proteins. The presence of the signature motif and the twelve transmembrane domains is consistent with NPTs belonging to the major facilitator superfamily of solute transporters. Note that *Pf*NPT1 and *Pb*NPT1 occur in syntenic positions on the *P.*

falciparum and *P. berghei* genomes, strong evidence that *Pb*NPT1 and *Pf*NPT1 are orthologues.

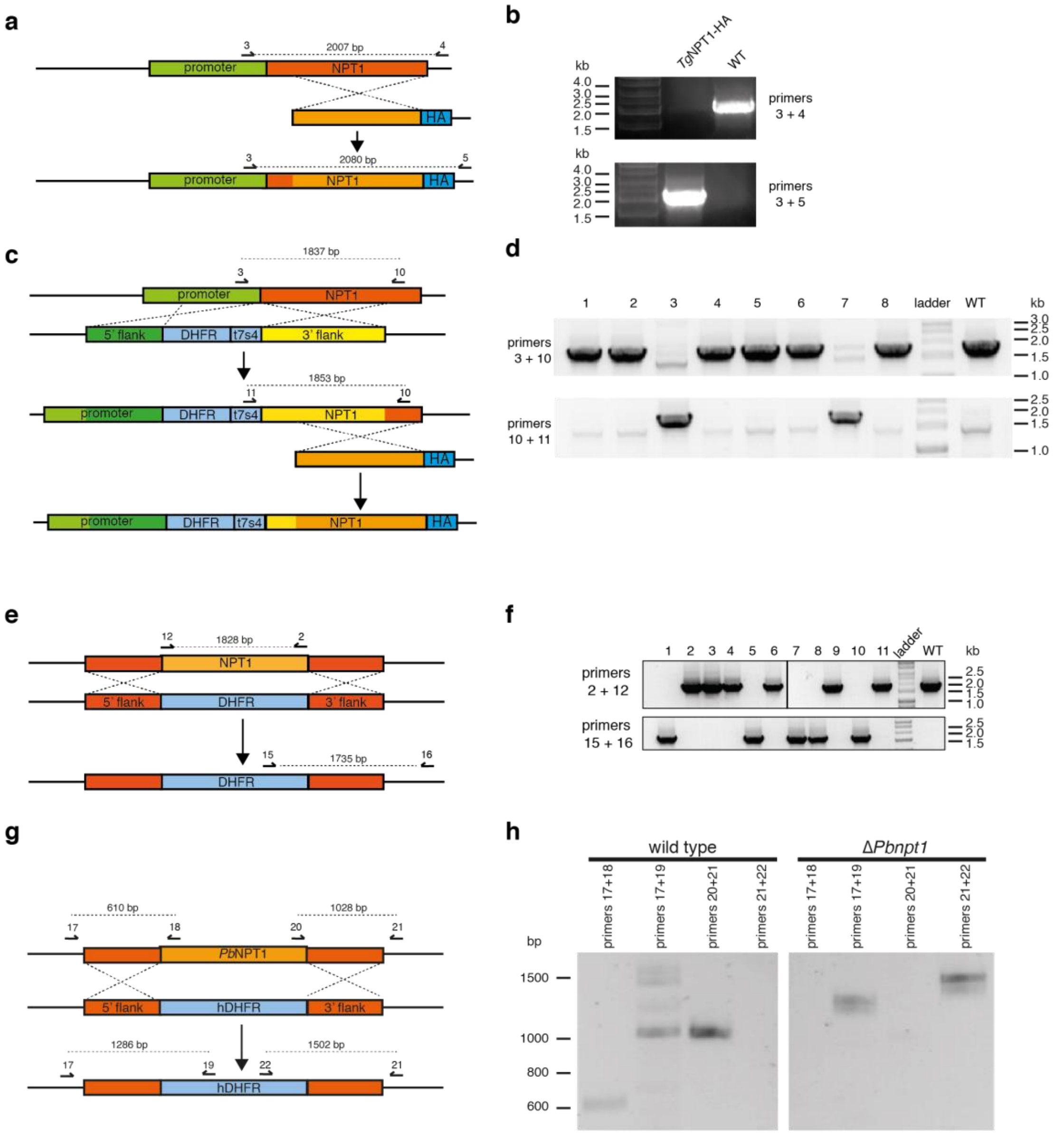

Supplementary Figure 2. Generation of parasite strains used in this study. (**a**) A schematic representation of the *Tg*NPT1 3’ HA-tag replacement strategy. The 3’ region of the native *Tg*NPT1 gene was replaced with a cassette containing a 1xHA tag by homologous recombination. Note that this figure is not drawn to scale. (**b**) PCR screen of the *Tg*NPT1-HA cell line following replacement of the 3’ region of *Tg*NPT1 with a HA tag. Genomic DNA from a clonal *Tg*NPT1-HA cell line and wild type RH strain (WT) were used for PCR analysis using primer pair 3+4 (to detect the presence of the native genomic locus) and primer pair 3+5 (to detect the modified locus). (**c**) A schematic representation of the *Tg*NPT1 promoter replacement strategy. The native *Tg*NPT1 promoter was replaced with an ATc-regulatable promoter, containing seven copies of the tet operator sequence and a *Tg*Sag4 minimal promoter (t7s4; [2]), by double homologous recombination. A pyrimethamine-resistant *T. gondii* dihydrofolate reductase (DHFR) gene was used as the selectable marker. The same cell line was later subjected to a second genetic manipulation to introduce a

hemagglutinin (HA) epitope at the 3'-end of the gene as described in Supplementary Fig. 2a. Note that this figure is not drawn to scale. (**d**) PCR screens of clonal cell lines following replacement of the *Tg*NPT1 promoter with the ATc-regulated promoter. Eight clones and the wild type parental cell line were screened using primer pairs 3+10 (to detect the presence of the native *Tg*NPT1 locus; top) and 10+11 (to detect the modified locus; bottom). The banding pattern of clones 3 and 7 indicated successful replacement of the *Tg*NPT1 promoter. Primers 3+10 are predicted to amplify a PCR product of 5468 bp in the modified locus, but this was not observed, probably because the PCR extension time was too short. (**e**) A schematic representation of the *TgNPT1* knockout strategy. The *Tg*NPT1 open reading frame was replaced with a pyrimethamine-resistant *T. gondii* dihydrofolate reductase (DHFR) gene by double homologous recombination. This figure is not drawn to scale. (**f**) PCR screens of clonal cell lines following *Tg*NPT1 knockout. Eleven clones and the wild type parental cell line were screened using primer pairs 2+12 (to detect a PCR product if the native *Tg*NPT1 locus was present; top) and 15+16 (to detect a PCR product if DHFR had successfully replaced *Tg*NPT1; bottom). The banding pattern of clones 1, 5, 7, 8 and 10 indicated successful replacement of the *Tg*NPT1 promoter. (**g**) A schematic representation of the gene replacement strategy for *Pb*NPT1 knockout. The wild type *Pb*NPT1 locus was replaced with human DHFR, a selectable marker that confers pyrimethamine resistance, by double homologous recombination. This figure is not drawn to scale. (**h**) Diagnostic PCRs to verify successful knockout of *Pb*NPT1. Genomic DNA extracted from wild type (left) and Δ*Pbnpt1* parasites (right) was screened for the presence of the 5' and 3' flanks of the native gene with primer pairs 17+18 and 20+21, respectively, and for the presence of the disrupted 5' and 3' flanks with primer pairs 17+19 and 21+22, respectively. Note that primer pair 17+19 gave non-specific PCR products when wild type DNA was used as template.

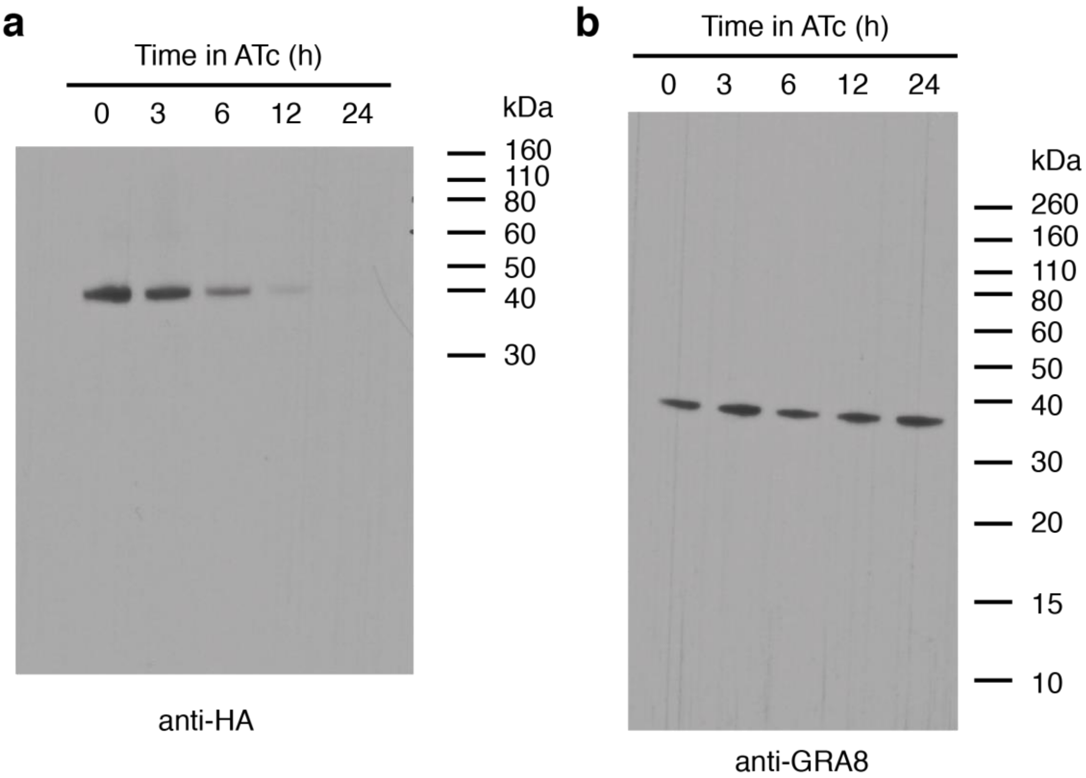

Supplementary Figure 3. Knockdown of *Tg*NPT1-HA in the i*Tg*NPT1-HA parasite strain. Western blot analysis demonstrating *Tg*NPT1-HA knockdown in the presence of ATc. Parasites were grown for 0, 3, 6, 12 and 24 hours in ATc and probed with (**a**) anti-HA to detect *Tg*NPT1-HA, or (**b**) GRA8 as a loading control. These are the unspliced blots of the data depicted in Fig. 1c of the manuscript.

Amino acid	mM	
	RPMI	DME
Glycine	0.133	0.4
Arginine	1.15	0.4
Asparagine	0.379	-
Aspartic acid	0.15	-
Cystine	0.208	0.2
Glutamic acid	0.136	-
Glutamine	2.05	4
Histidine	0.0968	0.2
Hydroxyproline	0.153	-
Isoleucine	0.382	0.8
Leucine	0.382	0.8
Lysine	0.219	0.8
Methionine	0.101	0.201
Phenylalanine	0.0909	0.4
Proline	0.174	-
Serine	0.286	0.4
Threonine	0.168	0.8
Tryptophan	0.0245	0.078
Tyrosine	0.111	0.423
Valine	0.171	0.8

higher
lower
similar
absent

Supplementary Figure 4. The amino acid composition of RPMI and DMEM. The amino acid composition of RPMI and DMEM is color-coded to indicate amino acids that are present at higher (blue), lower (yellow) or equivalent (lilac) levels in DMEM compared to RPMI. Amino acids absent in DMEM but present in RPMI are labelled in red.

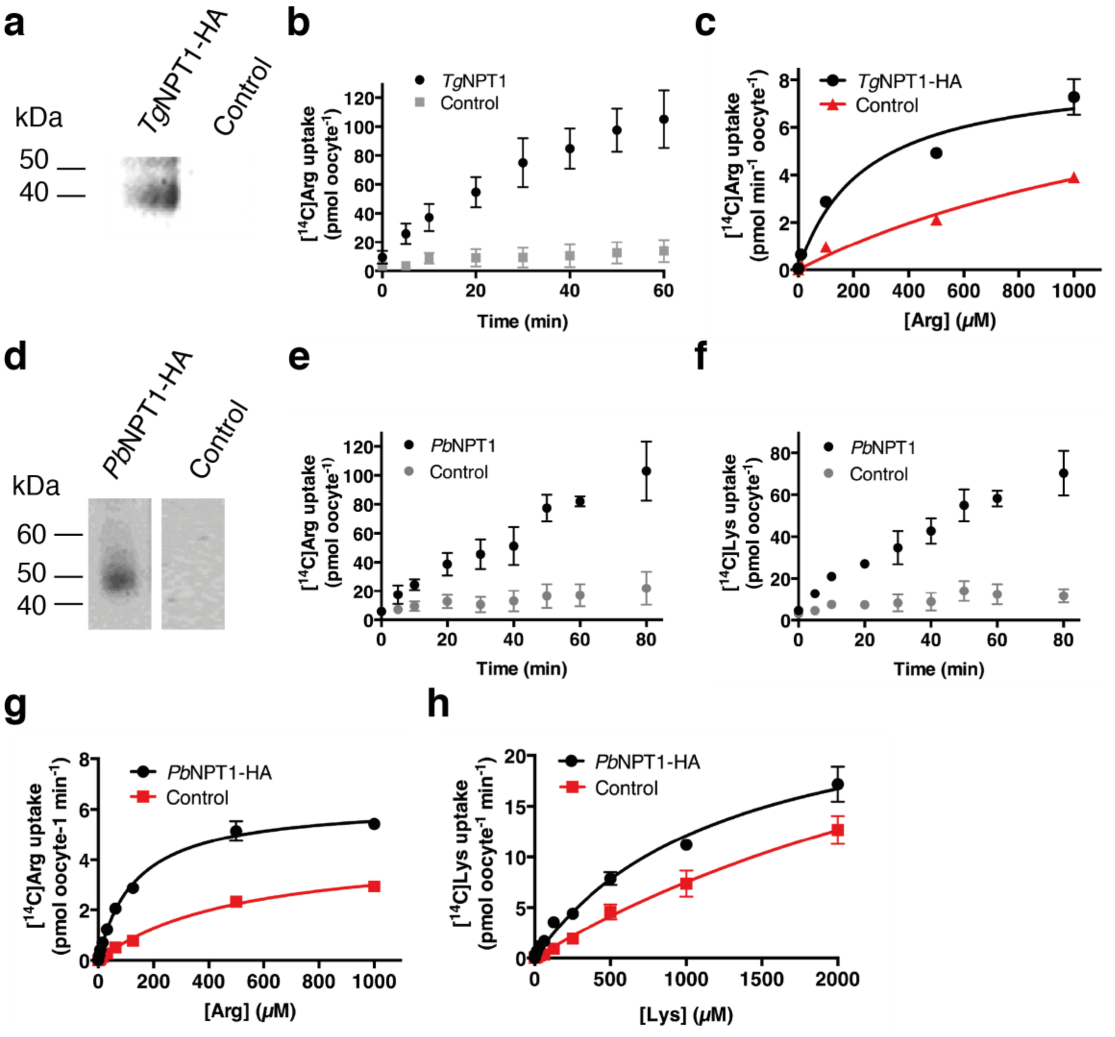

Supplementary Figure 5. Characterisation of *Tg*NPT1 and *Pb*NPT1 proteins expressed in *X. laevis* oocytes. (**a**) Western blot analysis of surface biotinylated proteins from oocytes expressing *Tg*NPT1-HA (left) or water-injected control oocytes (right), probed with anti-HA antibody to detect *Tg*NPT1-HA. Five to ten oocytes were processed for each condition and a protein amount equivalent to that from one oocyte was loaded onto each lane. (**b**) A time course for the uptake of [^{14}C]Arg into *X. laevis* oocytes expressing *Tg*NPT1-HA (black) or into uninjected oocytes (grey; control). Uptake was measured over 60 min in the presence of 100 µM unlabeled arginine and 289 nM [^{14}C]Arg. Each data point represents the mean uptake averaged from three independent experiments and are shown ± S.E.M. (**c**) Concentration-dependence of arginine transport in oocytes expressing *Tg*NPT1-HA (black) and in uninjected oocytes (red). These are the raw data that form the basis of Fig. 3b, in which uptake into uninjected oocytes (control) at each of the concentrations tested was subtracted from the uptake into *Tg*NPT1-expressing oocytes, yielding the *Tg*NPT1-mediated uptake component. The data are averaged from three experiments, each conducted on oocytes from a different frog, and are shown ± SEM (**d**) Western blot analysis of surface biotinylated proteins from oocytes expressing *Pb*NPT1-HA (left) or water-injected oocytes (right), probed with anti-HA antibody to detect *Pb*NPT1-HA. Both lanes represent images from the same membrane and exposure time, but have been spliced for easier interpretation. (**e-f**) A time course for [^{14}C]Arg (e) and [^{14}C]Lys (f) uptake into *X. laevis* oocytes expressing *Pb*NPT1-HA (black) or into uninjected

oocytes (grey; control). Uptake was measured over 80 min in the presence of 100 μM unlabelled arginine and 289 nM [^{14}C]Arg (e), or 100 μM unlabelled lysine and 307 nM [^{14}C]Arg. Each data point represents the mean uptake averaged from three independent experiments and are shown ± S.E.M. (**g-h**) Concentration-dependence of arginine (g) and lysine (h) uptake into oocytes expressing *Pb*NPT1-HA (black) and into uninjected oocytes (red; control). These are the raw data that form the basis of Fig. 8c-d, in which uptake into uninjected oocytes at each of the concentrations tested was subtracted from the uptake into *Pb*NPT1-expressing oocytes, yielding the *Pb*NPT1-mediated uptake component. The data are averaged from that obtained in three independent experiments, each conducted on oocytes from a different frog, and are shown ± SEM.

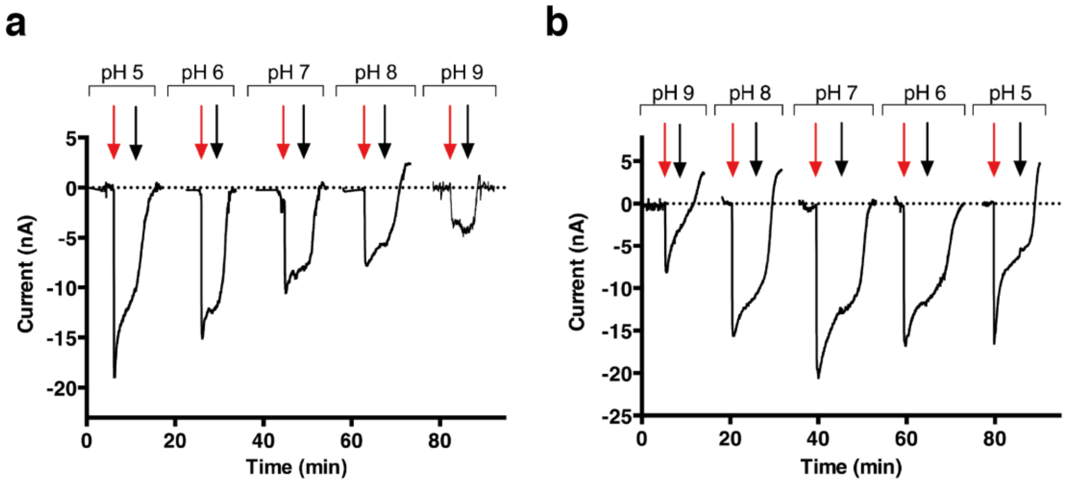

Supplementary Figure 6. Two-electrode voltage clamp recordings of oocytes expressing *Tg*NPT1-HA and exposed to media at pH 5-9. Panels (a) and (b) show representative current traces obtained using oocytes from two different frogs. The oocytes were voltage clamped at -50 mV and exposed to media with different pH values. The red arrows indicate the points of addition of 5 mM arginine to the media; the black arrows indicate the points at which the arginine was removed from the media.

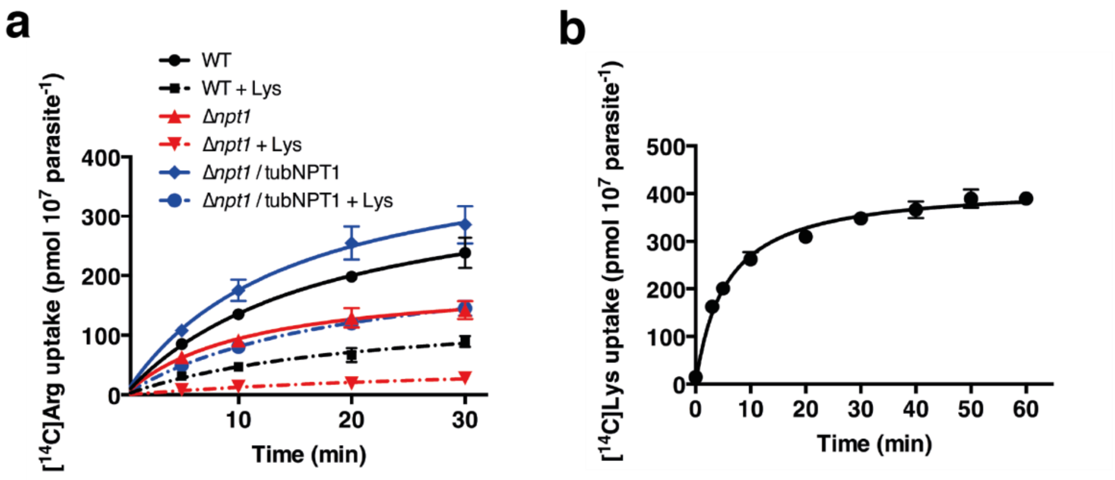

Supplementary Figure 7. Time courses for the uptake of [^{14}C]Arg and [^{14}C]Lys in *T. gondii*. (**a**) [^{14}C]Arg uptake into WT parasites (black), Δ*npt1* parasites (red), and Δ*npt1*/tubNPT1 parasites (blue) in the absence (solid line) or presence (dotted lines) of 80 μM unlabelled lysine. The parasites were suspended in PBS containing 10 mM glucose, 40 μM unlabelled arginine and 0.1 μCi/ml (289 nM) [^{14}C]Arg. The initial rates of [^{14}C]Arg transport presented in Fig. 5a were derived from the initial slopes of the fitted (single-order exponential) curves. (**b**) [^{14}C]Lys uptake into WT parasites, measured over 60 min. The parasites were suspended in PBS containing 10 mM glucose, 50 μM unlabelled lysine and 0.1 μCi/ml (307 nM) [^{14}C]Lys. For both (a) and (b) the data represent the mean ± SEM from three independent experiments.

Supplementary Table 1. A list of oligonucleotides used in this study.

No.	Primer name	Primer sequence (5'–3')
1	*Tg*NPT1 3'rep fwd	GATCGGATCCCAGTCGTTGTTCACTATTGCCATG
2	*Tg*NPT1 3'rep rvs	GACTCCTAGGTGCTGCATCGGCAGCGTGTGCT
3	*Tg*NPT1 screen fwd	GACTTTAGCCTTTCTCGGTCCG
4	*Tg*NPT1 screen rvs 1	CCAACTGTTTCTGCATCGTCGT
5	*Tg*DHFR 3'utr rvs	GTCATCCCTTTTCTTCGATAA
6	*Tg*NPT1 3'flank fwd	GATCCCCGGGATGGCGGGTCTGCTTAGCTCGT
7	*Tg*NPT1 3'flank rvs	GACTGCGGCCGCTTCTTCCGATTCCTTTTTGTCCT
8	*Tg*NPT1 5'flank fwd	GACTTTAATTAATAGCACAGGACGAGAAAAGTGTC
9	*Tg*NPT1 5'flank rvs	GATCGGCCGGCCAATGCCAACACGAATGAGATTCAACAG
10	*Tg*NPT1 screen rvs 2	CTTTGAGTTCCAGCACGCGACTG
11	t7s4 forward	ACGCAGTTCTCGGAAGACG
12	*Tg*NPT1 cDNA for	GATCGGATCCAAAATGGCGGGTCTGC
13	Δ*Tg*NPT1 3'flank fwd	GATCACTAGTACATCCACATGGCGATGCATTGGGTG
14	Δ*Tg*NPT1 3'flank rvs	GATCGCGGCCGCCGAGGCTGGTATCAAACGGTAAGGC
15	DHFR fwd screen	GAGTGCTGGACTGTTGCTGTCTGC
16	Δ*npt1* screen rvs	GCCCACGTATAGTGTAATGGAGAAGG
17	*Pb*NPT1 5' flank fwd	TTGACAAAAAAAATGAAGAGTTACC
18	*Pb*NPT1 5' flank rvs	ATGGTGTTTTTTGTTTTGCTCC
19	hDHFR 5' rvs	CAGGAATGGAGAACCAGGTCTT
20	*Pb*NPT1 3' flank fwd	GTTTTTACCACTAAGCATTTTATATACTAG
21	*Pb*NPT1 3' flank rvs	GGCTTATAGTATGTTAAATTTGAACG
22	hDHFR 3' fwd	GAAATATAAACTTCTGCCAGAATACC
23	*Tg*NPT1 oocyte fwd	GATCCCCGGCCACCATGGCGGGTCTGCTTAGCTCGT
24	*Tg*NPT1 oocyte rvs	GATCTCTAGACTAAGCGTAGTCCGGGACATCGTACGGG
25	ch*Pb*NPT1 fwd	TGTCATCCCGGGGCCACCATG
26	ch*Pb*NPT1 rvs	GGAACTCCTAGGGGCCTGAATCT

Supplementary References

1. Pao, S.S., Paulsen, I.T. & Saier, M.H., Jr. Major facilitator superfamily. *Microbiol Mol Biol Rev* **62**, 1-34 (1998).
2. Meissner, M., Schluter, D. & Soldati, D. Role of *Toxoplasma gondii* myosin A in powering parasite gliding and host cell invasion. *Science* **298**, 837-840 (2002).
